# Combining Isotretinoin and Topical Cholesterol/Atorvastatin in the Treatment of Linear Porokeratosis: A Case Report

**DOI:** 10.7759/cureus.38873

**Published:** 2023-05-11

**Authors:** Abdullah Alakeel, Sakhr Dawari, Ahmed Alhumidi, Khalid Alekrish

**Affiliations:** 1 Department of Dermatology, College of Medicine, King Saud University, Riyadh, SAU; 2 Department of Dermatology, Armed Forces Hospitals Southern Region, King Fahad Military Hospital, Khamis Mushait, SAU; 3 Department of Pathology, College of Medicine, King Saud University, Riyadh, SAU

**Keywords:** report, porokeratosis, linear, isotretinoin, atorvastatin

## Abstract

Linear porokeratosis (LP) is an epidermal keratinization disorder manifesting in the form of annular plaques with an atrophic center and hyperkeratotic margins. Although rare, LP carries a significant risk of skin cancer. Histological examination usually reveals the cornoid lamella, a parakeratosis column visualized in the outer layer of the epidermis. First-line treatment of LP is retinoids. However, the effects of combination therapy of isotretinoin and topical statins on LP are not well-understood. Herein, we attempted treatment with both isotretinoin and 2% cholesterol/atorvastatin ointment, with considerable improvement observed using the former but not the latter. These findings suggest that 2% topical cholesterol/atorvastatin treatment may not carry any additional benefits, even if used alongside retinoids. Further studies are needed to assess the potential effects of statins on LP.

## Introduction

Isidor Neumann, in the year 1875, was the first person to describe the condition known as porokeratosis (PK) [1]. Its classification is generally morphology-based, with porokeratosis of Mibelli, disseminated superficial porokeratosis, porokeratosis palmaris et plantaris disseminata, disseminated superficial actinic porokeratosis (DSAP), and linear porokeratosis (LP) being the most well-recognized types in the medical literature [2]. Epidermal keratinization manifesting in the form of annular plaques with an atrophic center and hyperkeratotic margins are the characteristic clinical features of PK [3]. Although not pathognomonic, the defining histological characteristic of porokeratosis is the cornoid lamella, a column of parakeratosis typically seen in the outer layers of the epidermis [3]. Regardless of its subtype, PK is generally considered a premalignant lesion [4]. Compared to other PK types, LP carries a higher risk of malignant transformation [5]. The risk of LP transformation into squamous cell or basal cell carcinoma motivates the search for more therapeutic advances.

It is generally agreed upon that retinoids, such as isotretinoin at a dose of 20 mg for about 20 weeks, are the first-line treatment for LP [6]. With inconsistent results, topical cholesterol/atorvastatin has also been explored as a therapy [7-9]. The effects of combining isotretinoin with topical cholesterol/atorvastatin as dual therapy have not been well studied. Given the inconsistent results of using cholesterol/atorvastatin in LP, we attempted treatment of LP with both isotretinoin and topical cholesterol/atorvastatin. If this therapy combination is successful, it may result in quicker remission of the rash, a reduction in isotretinoin dosage used for LP, or both. This study aimed to determine if adding topical cholesterol/atorvastatin to an ongoing course of isotretinoin while maintaining the same dosage resulted in accelerated rash clearance.

## Case presentation

A 14-year-old female with no previous medical history visited our dermatology clinic. The patient reported having several skin lesions on her left lower limb since the age of three months. At that time, it was a single lesion on the left knee, but it began to enlarge until the age of nine, when it progressed to involve the left knee and thigh. It was associated with frequent itching, and topical ointments only marginally alleviated itching. The patient denied having specific lesion triggers or a history of atopic dermatitis, asthma, food allergies, or joint pain. Moreover, the patient had no family history of similar symptoms, psoriasis, or genetic diseases. Apart from topical emollients, no previous treatment has been attempted.

**Figure 1:**
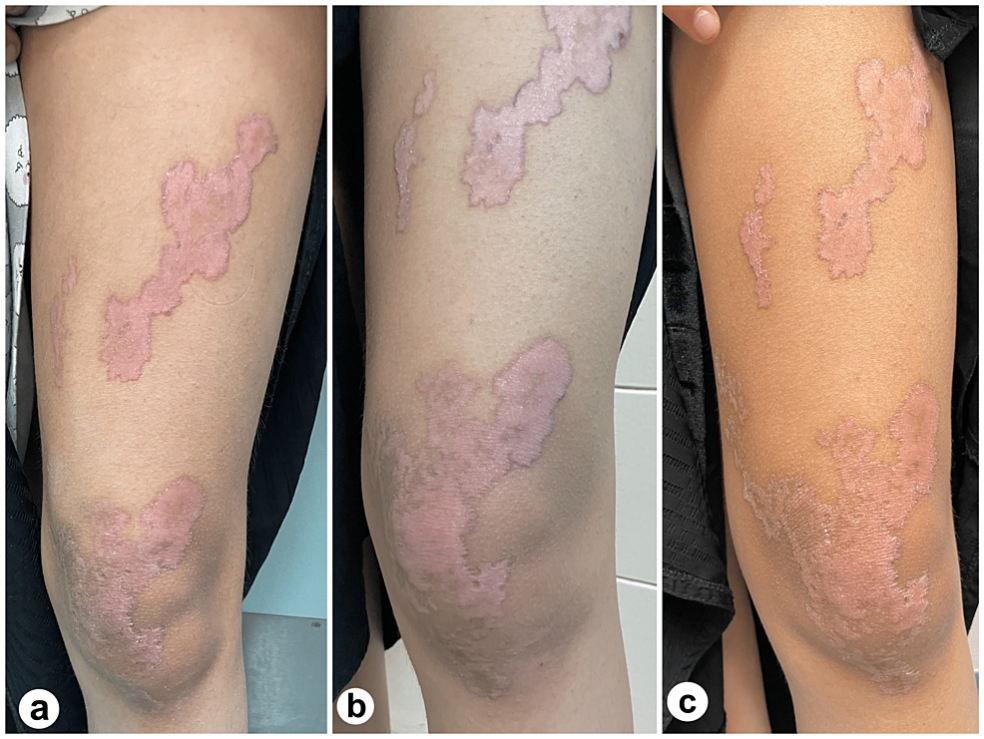
The left lower limb shows a few irregular linear and annular erythematous plaques, with a well-defined fine, scaly, and hyperpigmented border, following Blaschko's lines. a. Lesion before treatment. b. Lesion after treatment with 20 mg of isotretinoin for 10 weeks. c. Lesion after treatment with 20 mg of isotretinoin for 20 weeks, along with cholesterol/atorvastatin 2% ointment twice a day for 10 weeks.

**Figure 2:**
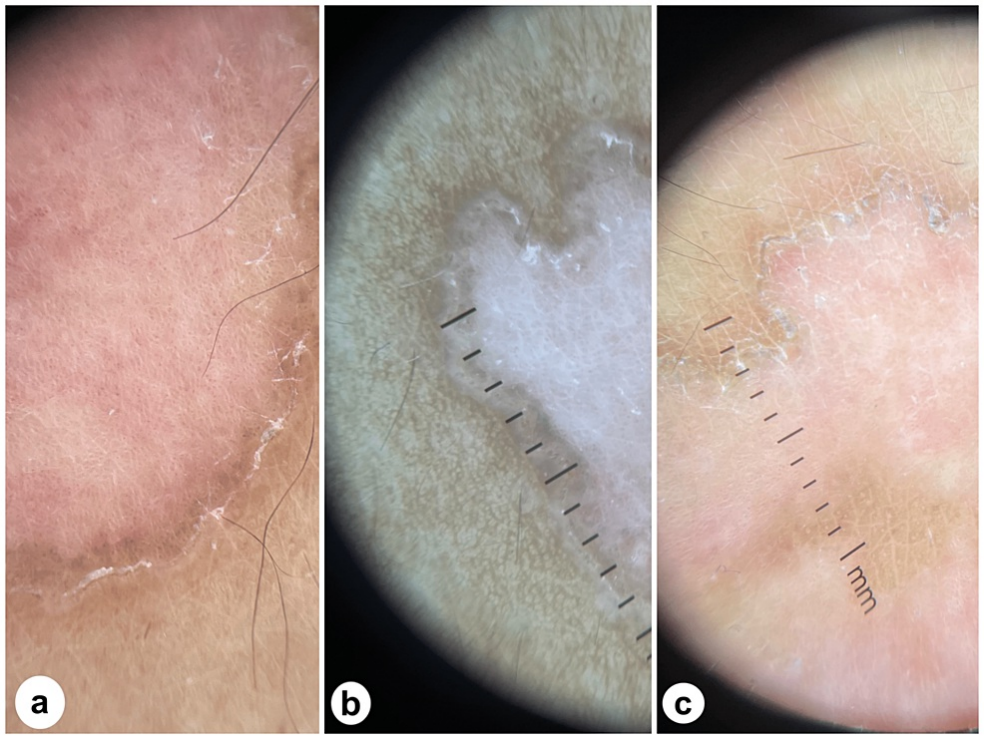
Dermatoscopy of the lesion showing the fine scaly border more clearly. a. Lesion appearance before treatment. b. Lesion appearance after treatment with 20 mg of isotretinoin for 10 weeks. c. Lesion appearance after treatment with 20 mg of isotretinoin for 20 weeks, along with cholesterol/atorvastatin 2% ointment twice a day for 10 weeks.

**Figure 3:**
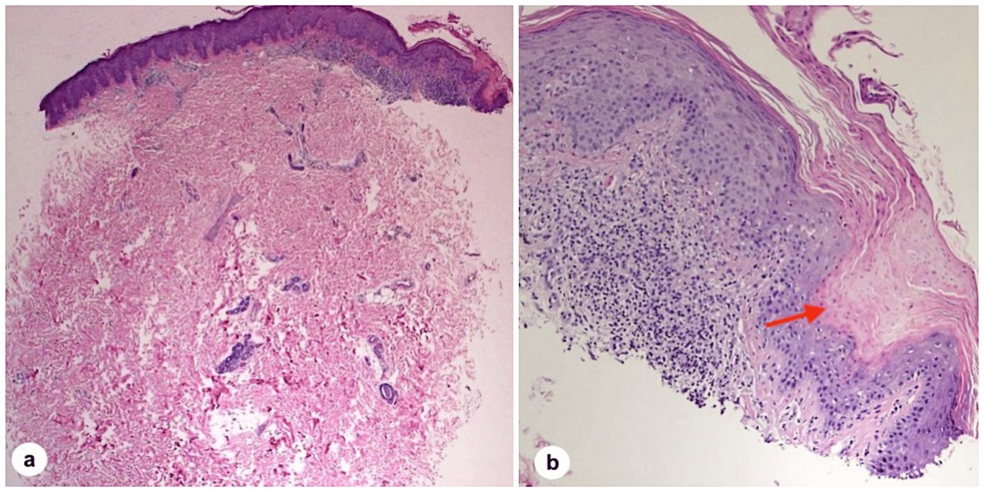
Histopathological analysis of the skin punch biopsy. a. Low magnification photograph showing acanthosis and focal parakeratosis (hematoxylin & eosin, X20). b. Higher magnification photograph showing the cornoid lamella (red arrow), a column of parakeratosis with underlying dyskeratosis (hematoxylin & eosin, X200).

On examination, the patient had a few irregular linear and annular erythematous plaques with a well-defined fine, scaly, and hyperpigmented border. These were located over the left knee and thigh and followed Blaschko's lines (Figure 1). The fine scaly border was more apparent upon dermatoscopic examination (Figure 2). Histopathological analysis is shown in Figure 3.

Based on the patient's medical history, characteristic physical examination findings, and histological findings, and after ruling out other differential diagnoses, the diagnosis of LP was established. Complete blood count, liver function test, lipid panel, and pregnancy test were all unremarkable. The patient was started on 20 mg of isotretinoin once a day, was educated about the importance of minimizing sun exposure and the neoplastic potential of the lesion, and a follow-up appointment was scheduled 10 weeks later.

Ten weeks later, the patient and her parents confirmed medication compliance, and apart from mild lip dryness, the treatment was well-tolerated. The liver function test and lipid panel were within the normal range. A significant reduction in itchiness has also been reported. Upon re-examination, a substantial improvement in the central erythema was noted, and there was no observed difference in rash size or the peripheral scale thickness (shown in Figures 1B, 2B). In conjunction with isotretinoin, 2% cholesterol/atorvastatin ointment was added twice daily to examine the benefit of this treatment combination in terms of time taken for rash clearance. Twenty weeks from treatment initiation, the itching did not improve from the first follow-up appointment. Unlike peripheral scales, peripheral lesion pigmentation improved by a small margin (shown in Figures 1C, 2C).

## Discussion

PK is a disorder of epidermal keratinization wherein annular plaques with an atrophic center and hyperkeratotic margins are its characteristic clinical features [3]. Its classification is generally morphology-based, with PM, DSAP, disseminated superficial porokeratosis, porokeratosis palmaris et plantaris disseminata, and LP being the most well-recognized types in the medical literature [2]. Although the clinical appearance of PK may vary significantly from patient to patient, the condition can be diagnosed through histological examination and visualization of the cornoid lamella. However, clinical correlation is paramount in such cases, given that the cornoid lamella is not considered pathognomonic and is found in various inflammatory and neoplastic skin pathologies, such as common warts, actinic keratosis, porokeratoma [4,10].

Benign lichenoid keratosis, a benign dermatological condition that manifests as a single papule or plaque, is another important differential diagnosis of PK and can pose difficulties in differentiating from PK. The distinction is predominantly established through a sufficient biopsy specimen that enables the visualization of the cornoid lamella. The differentiation between PK and lichenoid ketosis is crucial due to the potential malignancy associated with PK, whereas lichenoid ketosis does not pose such a risk [11].

As with all types of PK, the risk of squamous cell carcinoma and, to a lesser extent, basal cell carcinoma should be considered, with particular concern for disseminated and linear variants of PK [5]. Ultraviolet light has been shown to exacerbate symptoms and, in the worst-case scenario, promote the neoplastic transformation of these lesions. Therefore an accurate diagnosis is critical to receive the best possible treatment and reduce the risk of neoplastic progression [12].

Retinoids, taken at a dosage of 20 mg per day for 16-24 weeks, are regarded as the first-line therapy for LP [4,6]. Despite this, less than 20% of patients who receive proper treatment achieve complete resolution [4]. The mechanism by which retinoids affect keratinization diseases is complex. Simply put, retinoids are thought to interfere with keratinocyte differentiation, preventing the disease from fully manifesting [13]. In our case, isotretinoin was administered for approximately 20 weeks. The most significant improvement, a reduction in central erythema (shown in Figure 1B, 2B), was noted only during the first 10 weeks of treatment with isotretinoin. The duration of treatment required to observe any treatment effects is consistent with that reported by other authors [14,15]. Since the scale thickness, lesion size, and number did not change from weeks 11 to 20, it may be reasonable to infer that if no progress is seen at the 10-week mark, it may not be as beneficial to continue isotretinoin treatment.

Whether topical statins benefit patients with LP is still debatable in the literature [7-9]. Cholesterol is an essential component of the extracellular matrix of the stratum corneum and is required for optimum keratinocyte function and differentiation. Atzmony et al. discovered that a loss-of-function mutation in the genes for phosphomevalonate kinase and mevalonate diphosphate decarboxylase, both of which are rate-limiting enzymes in the mevalonate synthesis pathway, induces porokeratosis [16]. The inactivation of these enzymes results in a deficiency of extracellular cholesterol and the accumulation of deleterious metabolites in the mevalonate pathway, precipitating premature keratinocyte apoptosis and PK. It is hypothesized that cholesterol/statin therapy restores the equilibrium of the mevalonate pathway by replenishing deficient cholesterol and preventing the accumulation of toxic metabolites, thereby promoting proper differentiation and function of keratinocytes [8,16].

In cases where topical statins are regarded as effective, they are usually used for four weeks, after which a visible reduction in rash size should be appreciated, and in six to eight weeks, the rash should have entirely resolved [8,9]. Our outcome with statin therapy varies from those mentioned earlier [7,8] since treatment with 2% atorvastatin ointment twice a day for 10 weeks resulted in no change in lesion number, size, or scale thickness (shown in Figure 1C, 2C).

It is less likely that treatment with isotretinoin and cholesterol/atorvastatin dual therapy hindered medication response, as both should theoretically improve LP lesions. Because the lesion is in a photo-protected site, sun exposure is also improbable. A more plausible explanation, however, is that topical statins may not impact LP lesions to the extent that the duration of treatment is reduced less than in earlier cases [8,9]. However, it is crucial to highlight that this was the outcome of therapy in a single patient, and the results cannot be generalized without more studies. In addition, cholesterol/atorvastatin may have resulted in satisfactory results if administered at the start of treatment. More controlled studies, including more than one patient, would better evaluate the effectiveness of topical statins when paired with isotretinoin in treating LP.

## Conclusions

To the best of our knowledge, this is the first case of LP in which both isotretinoin and topical cholesterol/atorvastatin treatments are attempted. Dermatologists, particularly pediatric dermatologists, should be vigilant for LP in young patients as diagnosing LP earlier can save numerous unnecessary appointments and, most significantly, aid in detecting and, hopefully, preventing the potential neoplastic progression of LP. Retinoids are the first-line treatment for LP, and topical statin treatment may not carry any additional benefits, even if used alongside retinoids. Further studies are needed to assess the potential effects of statins on LP.
